# Crystal-structure determination and Hirshfeld surface analysis of two new thio­phene derivatives: (*E*)-*N*-{2-[2-(benzo[*b*]thio­phen-2-yl)ethen­yl]-5-fluoro­phen­yl}benzene­sulfonamide and (*E*)-*N*-{2-[2-(benzo[*b*]thio­phen-2-yl)ethen­yl]-5-fluoro­phen­yl}-*N*-(but-2-yn-1-yl)benzene­sulfonamide

**DOI:** 10.1107/S2056989023006096

**Published:** 2023-07-21

**Authors:** S. Madhan, M. NizamMohideen, Vinayagam Pavunkumar, Arasambattu K. MohanaKrishnan

**Affiliations:** a Department of Physics, The New College, Chennai 600 014, University of Madras, Tamil Nadu, India; b Department of Organic Chemistry, University of Madras, Guindy Campus, Chennai-600 025, Tamilnadu, India; Venezuelan Institute of Scientific Research, Venezuela

**Keywords:** crystal structure, thio­phene, benzo­thio­phene, flurophen­yl, phenyl­sulfonamide hydrogen bonding, Hirshfeld surface analysis

## Abstract

In the title compounds, C_22_H_16_FNO_2_S_2_ (**I**) and C_26_H_20_FNO_2_S_2_ (**II**), the benzo­thio­phene rings are essentially planar with maximum deviations of 0.009 (1) and 0.001 (1) Å for the carbon and sulfur atom in compounds **I** and **II**, respectively. In **I**, the thio­phene ring system is almost orthogonal to the phenyl ring attached to the sulfonyl group, with a dihedral angle of 77.7 (1)°. In compound **I**, the mol­ecular structure is stabilized by weak C—H⋯O intra­molecular inter­actions formed by the sulfone oxygen atoms, which generate two *S*(5) ring motifs. In the crystal of **I**, N—H⋯O hydrogen bonds link the mol­ecules into *R* ^2^_2_(8) rings, which are connected into a *C*(10) chain *via* C—H⋯F hydrogen bonds. Inter­molecular C—H⋯π inter­actions are also observed. In compound **II**, the mol­ecules are linked *via* C—H⋯O and C—H⋯F hydrogen bonding, generating infinite *C*(11) and *C*(13) chains running parallel to [010].

## Abstract 2

The crystal structures of two benzo­thio­phene derivatives are described and the inter­molecular contacts in the crystals analysed using Hirshfeld surface analysis and two-dimensional fingerprint plots.

## 1. Chemical context

Thio­phene, C_4_H_4_S, belongs to a class of aromatic five-membered heterocycles comprising one S heteroatom. Thio­phene derivatives possess pharmacological and biological activities including anti­bacterial (Mishra *et al.*, 2012 ▸), anti­allergic (Gillespie *et al.*, 1985 ▸), anti-cancer and anti-toxic (Gewald *et al.*, 1966 ▸), analgesic (Laddi *et al.*, 1998 ▸; Chen *et al.*, 2008 ▸), anti-inflammatory (Ferreira *et al.*, 2006 ▸), anti­oxidant (Jarak *et al.*, 2005 ▸), anti­tumor (Gadad *et al.*, 1994 ▸), anti­microbial (Abdel-Rahman *et al.*, 2003 ▸), anti­hypertensive (Monge Vega *et al.*, 1980 ▸), anti-diabetes mellitus (Abdelhamid *et al.*, 2009 ▸), gonadotropin releasing hormone antagonist (Sabins *et al.*, 1944 ▸) and they are building blocks in many agrochemicals (Ansary & Omar, 2001 ▸). Thio­phene possesses promising pharmacological activities, such as anti-HIV PR inhibitor (Bonini *et al.*, 2005 ▸) and anti-breast cancer (Brault *et al.*, 2005 ▸). Benzo­thio­phenes are biologically energetic mol­ecules. One of the most significant drugs based on the benzo­thio­phene structure is Raloxifine, used to treat osteoporosis in postmenopausal women (Jordan, 2003 ▸). Benzo­thio­phenes are also present in luminescent components used in organic materials (Russell & Press, 1996 ▸). Thio­phene derivatives have a wide variety of applications in optical and electronic systems (Gather *et al.*, 2008 ▸; He *et al.*, 2009 ▸) and are used extensively in solar cells (Justin Thomas *et al.*, 2008 ▸), organic light-emitting diodes (OLEDs) (Mazzeo *et al.*, 2003 ▸), organic field-effect transistors (OFETs) (Zhan *et al.*, 2007 ▸) and as NLO devices (Bedworth *et al.*, 1996 ▸; Raposo *et al.*, 2011 ▸). Thieno-pyridine products are used in medicine as allosteric adenosine receptors and in the treatment of adenosine-sensitive cardiac arrhythmias (Tumey *et al.*, 2008 ▸; Grunewald *et al.*, 2008 ▸). Recognizing the importance of such compounds in drug discovery and our ongoing research into the construction of novel thio­phene has prompted us to investigate the title thio­phene derivatives and we report herein their synthesis, crystal structures and Hirshfeld surface analysis. 
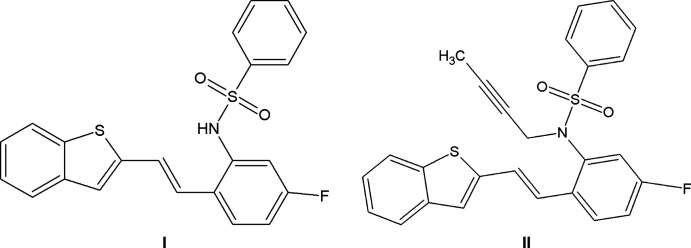


## 2. Structural commentary

**Figure 1:**
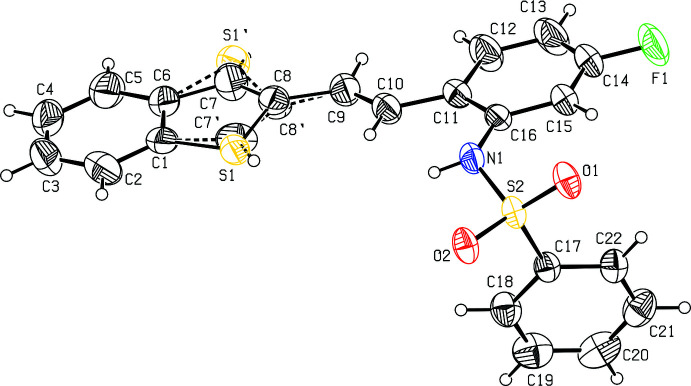
The mol­ecular structure of compound **I**, with atom labelling. Displacement ellipsoids are drawn at the 30% probability level.

**Figure 2:**
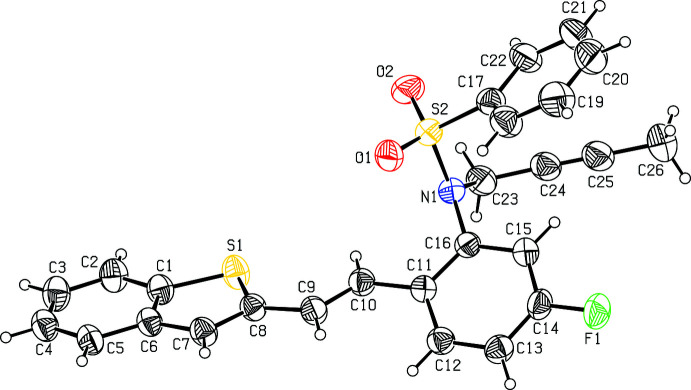
The mol­ecular structure of compound **II**, with atom labelling. Displacement ellipsoids are drawn at the 30% probability level.

The mol­ecular structure of compound **I**, C_22_H_16_FNO_2_S_2_ (Fig. 1 ▸), comprises a benzo­thio­phene ring system (S1/C1–C8) attached to an *N*-(5-fluoro-2-vinyl­phen­yl)benzene­sulfonamide (C7–C22/N1/S2/O1/O2/F1) while compound **II**, C_26_H_20_FNO_2_S_2_ (Fig. 2 ▸), comprises a benzo­thio­phene ring system (S1/C1–C8) attached to an *N*-but-2-yn-1-yl-*N*-(5-fluoro-2-vinyl­phen­yl)benzene­sulfonamide (C9–C15/N1/S2/O1/O2). In both compounds, the benzo­thio­phene ring systems (S1/C1–C8) are essentially planar with maximum deviations of 0.009 (1) and 0.001 (1) Å for atom C8 and S1 in compounds **I** and **II**, respectively. The mean planes of the thio­phene ring system in **I** make dihedral angles of 1.2 (2), 2.3 (2), 77.7 (2)° with the C1–C6, C11–C16 and C17–C22 phenyl rings. The mean planes of the thio­phene ring system in **II** make dihedral angles of 0.3 (2), 33.3 (2), 25.2 (2)°, respectively, with the C1–C6, C11–C16 and C17–C22 phenyl rings, The benzo­thio­phene ring system in **I** is almost orthogonal to the C17–C22 ring attached to sulfonyl group with dihedral angle of 77.7 (1)° in **I**. For both compounds, the bond lengths and angles are close to those observed for similar structures (Madhan *et al.*, 2022 ▸, 2023 ▸).

In both compounds, the tetra­hedral configuration is distorted around atom S1. The increase in the O2—S2—O1 angle [118.64 (9) in **I** and 120.6 (2)° in **II**], with a simultaneous decrease in the N1—S2—C17 angle [106.35 (9) in **I** and 108.3 (2)° in **II**] from the ideal tetra­hedral value (109.5°) are attributed to the Thorpe–Ingold effect (Bassindale, 1984 ▸). The widening of the angles may be due to the repulsive inter­action between the two short S=O bonds. In compound **II**, the N1—C23 = 1.483 (6) and N1—C16 = 1.450 (5) Å bond lengths in the mol­ecule are longer than the mean N*sp* ^2^—C*sp* ^2^ bond-length value of 1.355 (14) Å [Allen *et al.*, 1987 ▸; Cambridge Structural Database (CSD), Version 5.37; Groom *et al.*, 2016 ▸]. The elongation observed may be due to the electron-withdrawing character of the phenyl­sulfonyl group. In compound **II**, the sum of the bond angles around N1 (354.1°) indicates *sp* ^2^ hybridization.

In compound (**I**), the mol­ecular structure is stabilized by weak C15—H15⋯O1 intra­molecular inter­actions formed by the sulfone oxygen atoms, which generate two *S*(5) ring motifs (Fig. 1 ▸).

## 3. Supra­molecular features

**Figure 3:**
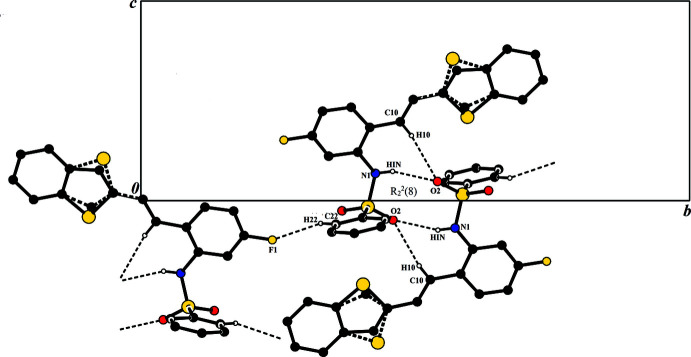
A view along the *a* axis of the crystal packing of compound **I**. The hydrogen bonds (Table 1 ▸) are shown as dashed lines, and H atoms not involved in hydrogen bonding have been omitted.

In the crystal of **I**, the C10—H10⋯O2^i^ hydrogen bond generates an inversion dimer with an 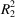 (14) ring motif; within the ring, N1—H1*N*⋯O2^ii^ hydrogen bonds link the mol­ecules into 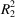 (8) ring motifs (Fig. 3 ▸ and Table 1 ▸). These rings are linked by the *C*(10) chain formed *via* the C22—H22⋯F1^iii^ hydrogen bonds. No significant C–H⋯π inter­actions with centroid distances of less than 4Å are observed in the structure.

**Figure 4:**
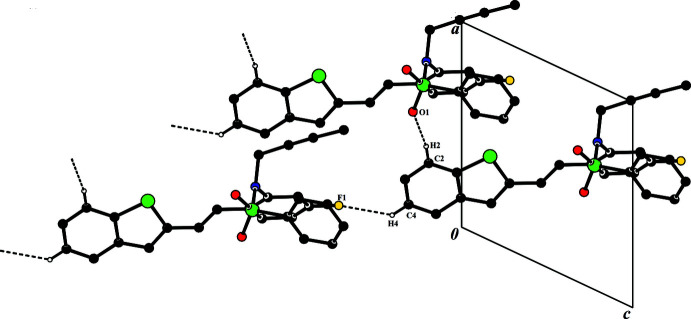
A view along the *b* axis of the crystal packing of compound **II**. The hydrogen bonds (Table 2 ▸) are shown as dashed lines, and H atoms not involved in hydrogen bonding have been omitted.

In thecrystal of **II**, mol­ecules are linked *via* C2—H2⋯O1^i^ and C4⋯H4⋯F1^ii^ inter­molecular hydrogen bonding, which generates infinite *C*(11) and *C*(13) chains running parallel to [010] (Bernstein *et al.*, 1995 ▸). In addition, the crystal packing features inter­molecular C—H⋯π (C23—H23*A*⋯*Cg*1^iii^) inter­actions, where the *Cg*1 is the centroid of the C1–C6 ring (Table 2 ▸, Fig. 4 ▸). No significant π⋯π inter­actions with inter­centroid distances of less than 4Å are observed in either structure.

## 4. Hirshfeld surface analysis

A recent article by Tiekink and collaborators (Tan *et al.*, 2019 ▸) reviews and describes the uses and utility of Hirshfeld surface analysis (Spackman & Jayatilaka, 2009 ▸), and the associated two-dimensional fingerprint plots (McKinnon *et al.*, 2007 ▸), to analyse inter­molecular contacts in crystals. The various calculations (*d_norm_*, curvedness and shape index and 2D fingerprint plots) were performed with *CrystalExplorer17* (Turner *et al.*, 2017 ▸).

**Figure 5:**
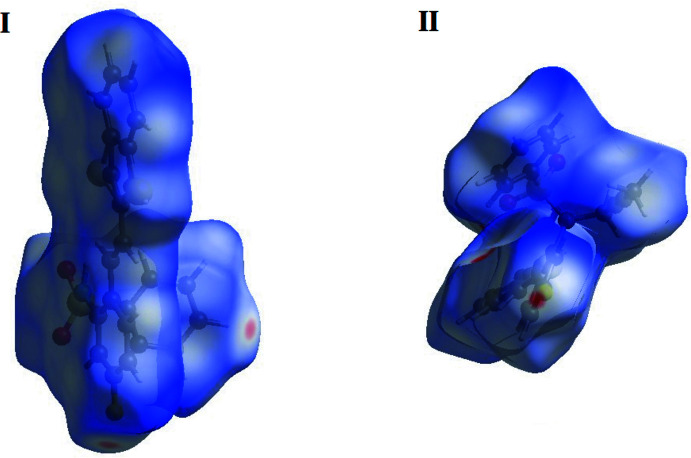
The Hirshfeld surfaces of compounds **I** and **II**, mapped over *d*_norm_.

**Figure 6:**
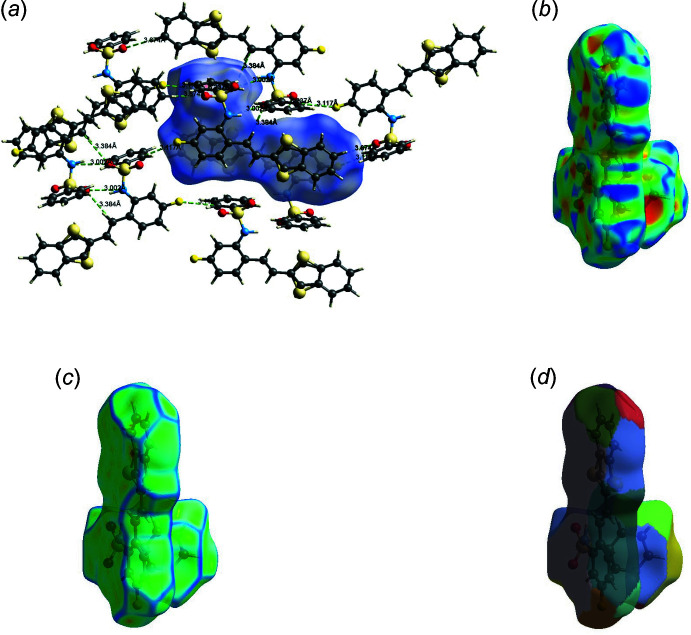
The Hirshfeld surfaces for visualizing the inter­molecular contacts of compound **I**: (*a*) *d_norm_* of compound **I**, showing the various inter­molecular contacts in the crystal, (*b*) shape index, (*d*) curvedness and (*e*) fragment patches.

**Figure 7:**
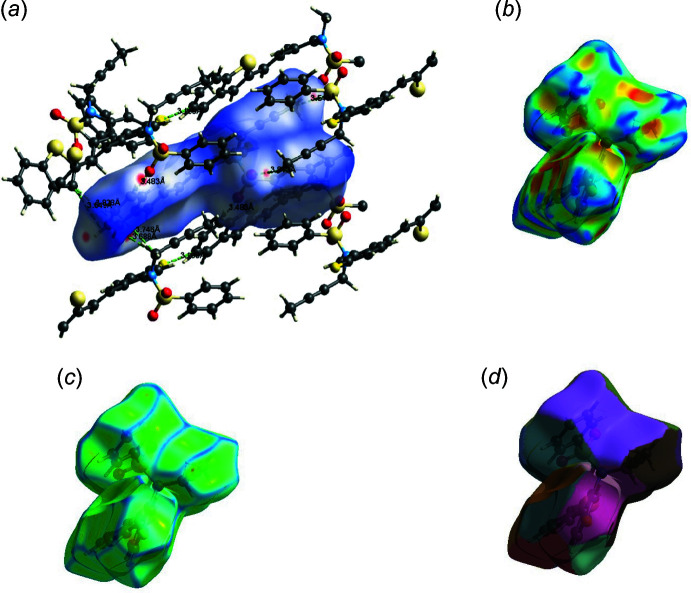
The Hirshfeld surfaces for visualizing the inter­molecular contacts of compound **II**: (*a*) *d_norm_* of compound **II**, showing the various inter­molecular contacts in the crystal, (*b*) shape index, (*d*) curvedness and (*e*) fragment patches.

The Hirshfeld surfaces of compounds **I** and **II** mapped over *d_norm_* are given in Fig. 5 ▸, and the inter­molecular contacts are illustrated in Fig. 6 ▸ *a* for **I** and Fig. 7 ▸ *a* for **II**. They are colour-mapped with the normalized contact distance, *d*_norm_, from red (distances shorter than the sum of the van der Waals radii) through white to blue (distances longer than the sum of the van der Waals radii). The *d*_norm_ surface was mapped over a fixed colour scale of −0.434 (red) to 1.449 (blue) for compound **I** and −0.119 (red) to 1.765 (blue) for compound **II**, where the red spots indicate the inter­molecular contacts involved in the hydrogen bonding. The electrostatic potential was also mapped on the Hirshfeld surface using a STO-3G basis set and the Hartee–Fock level of theory (Spackman *et al.*, 2008 ▸; Jayatilaka *et al.*, 2005 ▸). The presence of inter­actions is indicated by a red and blue colour on the shape-index surface (Fig. 6 ▸ *b* for **I** and 7*b* for **II**). Areas on the Hirshfeld surface with high curvedness tend to divide the surface into contact patches with each neighbouring mol­ecule. The coordination number in the crystal is defined by the curvedness of the Hirshfeld surface (Fig. 6 ▸ *c* for **I** and Fig. 7 ▸ *c* for **II**). The nearest neighbour coordination environment of a mol­ecule is identified from the colour patches on the Hirshfeld surface depending on their closeness to adjacent mol­ecules (Fig. 6 ▸ *d* for **I** and Fig. 7 ▸ *d* for **II**).

**Figure 8:**
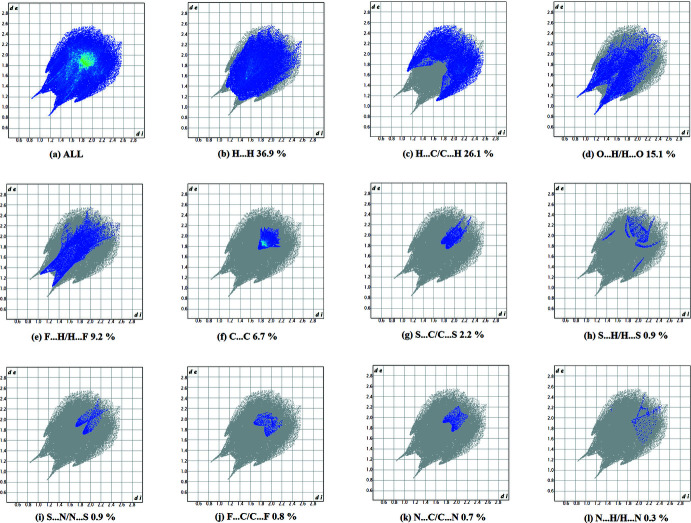
The full two-dimensional fingerprint plot for compound **I**, and fingerprint plots delineated into (*b*) H⋯H, (*c*) O⋯H/H⋯O, (*d*) C⋯H/H⋯C, (*e*) C⋯C and (*f*) N⋯H/H⋯N contacts.

The fingerprint plots are given in Figs. 8 ▸ and 9 ▸. For compound **I**, they reveal that the principal inter­molecular contacts are H⋯H contacts at 36.9% (Fig. 8 ▸ *b*), H⋯C/C⋯H contacts at 26.1% (Fig. 8 ▸ *c*), O⋯H/H⋯O at 15.1% (Fig. 8 ▸ *d*), F⋯H/H⋯F at 9.2% (Fig. 8 ▸ *e*), C⋯C at 6.7% (Fig. 8 ▸ *f*), S⋯C/C⋯S at 2.2% (Fig. 8 ▸ *g*), S⋯H/H⋯S contacts at 0.9% (Fig. 8 ▸ *i*), F⋯C/C⋯F at 0.8% (Fig. 8 ▸ *j*), N⋯C/C⋯N at 0.7% (Fig. 8 ▸ *k*) and N⋯H/H⋯N contacts at 0.3% (Fig. 8 ▸ *l*).

**Figure 9:**
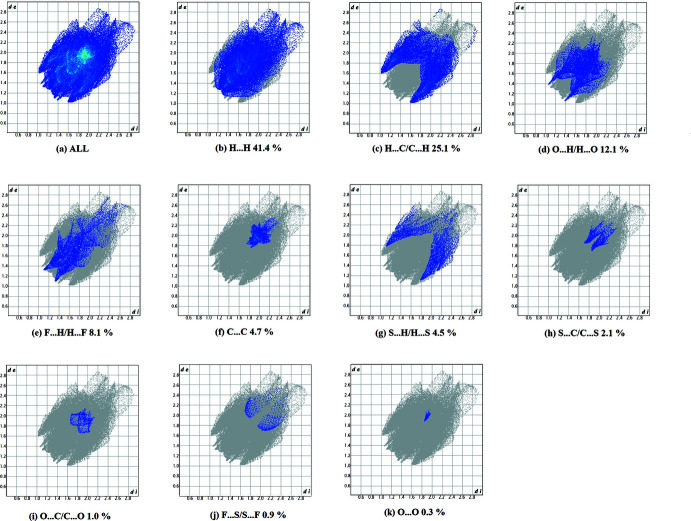
The full two-dimensional fingerprint plot for compound **II**, and fingerprint plots delineated into (*b*) C⋯C, (*c*) C⋯H/H⋯C, (*d*) C⋯·N/N⋯C, (*e*) C⋯O/O⋯C, (*f*) H⋯H, (*g*) N⋯H/H⋯N, (*h*) O⋯H/H⋯O and (I) S⋯H/H⋯S contacts.

For compound **II**, they reveal a similar trend, with the principal inter­molecular contacts being H⋯H/H⋯H at 41.4% (Fig. 9 ▸ *b*), H⋯C/C⋯H contacts at 25.1% (Fig. 9 ▸ *c*), O⋯H/H⋯O at 12.1% (Fig. (9*d*), F⋯H/H⋯F at 8.1% C⋯C at 4.6% (Fig. 9 ▸ *e*), C⋯·C at 4.7% (Fig. 9 ▸ *f*), S⋯H/H⋯S contacts at 4.5% (Fig. 9 ▸ *g*), S⋯C/C⋯S contacts at 2.1% (Fig. 9 ▸ *h*), C⋯O/O⋯C contacts at 1.0% (Fig. 9 ▸ *i*), F⋯S/S⋯F at 0.9% (Fig. 9 ▸ *j*) and O⋯O contacts at 0.3 (Fig. 9 ▸ *k*). In both compounds, the H⋯H inter­molecular contacts predominate, followed by the C⋯H/H⋯C and O⋯H/H⋯O contacts.

## 5. Synthesis and crystallization

Compound **I**: To a solution of (*E*)-2-(2-(benzo[*b*]thio­phen-2-yl)vin­yl)-5-fluoro­benzenaminium chloride (1.2 g, 3.934 mmol) in dry DCM (10 mL), pyridine (0.47 mL, 5.901 mmol) and PhSO_2_Cl (0.6 mL, 4.721 mmol) were added and stirred at room temperature for 12 h. After completion of the reaction (monitored by TLC), it was poured into crushed ice (50 g) containing conc. HCl (5 mL), extracted with DCM (2 × 20 mL) then washed with water (2 × 20 mL) and dried (Na_2_SO_4_). Removal of solvent *in vacuo* followed by crystallization from di­ethyl­ether (4 mL) afforded (*E*)-*N*-{2-[2-(benzo[*b*]thio­phen-2-yl)ethen­yl]-5-fluoro­phen­yl}benzene­sulf­onamide as a white solid.

Compound **II**: To a solution of (*E*)-*N*-{2-[2-(benzo[b]thio­phen-2-yl)vin­yl]-5-fluoro­phen­yl}benzene­sulfonamide (0.70 g, 1.711 mmol) in CH_3_CN (10 mL), K_2_CO_3_ (0.35 g, 2.567 mmol) and 1-bromo­but-2-yne (0.22 mL, 2.567 mmol) were added and stirred at room temperature for 12 h. After completion of the reaction (monitored by TLC), it was poured into crushed ice (50 g) containing conc. HCl (5 mL), extracted with ethyl acetate (2 × 20 mL) then washed with water (2 × 20 mL) and dried (Na_2_SO_4_). Removal of solvent *in vacuo* followed by crystallization from methanol (4 mL) afforded (*E*)-*N*-{2-[2-(benzo[*b*]thio­phen-2-yl)ethen­yl]-5-fluoro­phen­yl}-*N*-(but-2-yn-1-yl)benzene­sulfonamide as a white solid.

## 6. Refinement

Crystal data, data collection and structure refinement details are summarized in Table 3 ▸. For compound **I**, the NH H atoms were located in difference-Fourier maps and freely refined. For compound **II**, they were included in calculated positions and refined as riding: N—H = 0.93 Å with *U*_iso_(H) = 1.2*U*_eq_(N). All C-bound H atoms were positioned geometrically and constrained to ride on their parent atoms: C–H = 0.93–0.97 Å with *U*_iso_(H) = 1.5*U*_eq_(C-meth­yl) and 1.2*U*_eq_(C) for other H atoms. In compound **I**, the thio­phene ring is disordered over two positions with a refined occupancy ratio of 0.756 (4):0.244 (3). The geometries were regularized using soft restraints.

## Supplementary Material

Crystal structure: contains datablock(s) global, I, II. DOI: 10.1107/S2056989023006096/zn2029sup1.cif

Structure factors: contains datablock(s) I. DOI: 10.1107/S2056989023006096/zn2029Isup2.hkl

Structure factors: contains datablock(s) II. DOI: 10.1107/S2056989023006096/zn2029IIsup3.hkl

Supporting information file. DOI: 10.1107/S2056989023006096/zn2029Isup4.cml

Supporting information file. DOI: 10.1107/S2056989023006096/zn2029IIsup5.cml

CCDC references: 2280606, 2280605

Additional supporting information:  crystallographic information; 3D view; checkCIF report

## Figures and Tables

**Table 1:** Hydrogen-bond geometry (Å, °) for **I** [Chem scheme1]

*D*—H⋯*A*	*D*—H	H⋯*A*	*D*⋯*A*	*D*—H⋯*A*
C15—H15⋯O1	0.93	2.30	2.980 (3)	130
C10—H10⋯02^i^	0.93	2.51	3.383 (3)	157
N1—H1*N*⋯O2^i^	0.83 (2)	2.21 (2)	3.001 (2)	161 (2)
C22—H22⋯F1^ii^	0.93	2.44	3.116 (3)	130

Symmetry codes: (i) 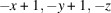 ; (ii) 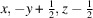 .

**Table 2:** Hydrogen-bond geometry (Å, °) for **II** [Chem scheme1] *Cg*1 is the centroid of the C1–C6 ring.

*D*—H⋯*A*	*D*—H	H⋯*A*	*D*⋯*A*	*D*—H⋯*A*
C2—H2⋯O1^i^	0.93	2.59	3.483 (7)	162
C4—H4⋯F1^ii^	0.93	2.52	3.188 (6)	130
C18—H18⋯S1^iii^	0.93	3.01	3.744 (6)	137
C23—H23*A*⋯*Cg*1^iv^	0.93	2.69	3.566 (6)	151

Symmetry codes: (i) 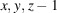 ; (ii) 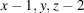 ; (iii) 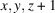 ; (iv) 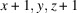 .

**Table 3:** Experimental details

	**I**	**II**
Crystal data		
Chemical formula	C_22_H_16_FNO_2_S_2_	C_26_H_20_FNO_2_S_2_
*M*_r_	409.48	461.55
Crystal system, space group	Monoclinic, *P*2_1_/*c*	Monoclinic, *P*2_1_/*c*
Temperature (K)	297	297
*a*, *b*, *c* (Å)	7.9588 (1), 25.9840 (4), 9.5178 (2)	9.3517 (3), 31.7075 (11), 8.6063 (3)
β (°)	96.853 (1)	115.179 (2)
*V* (Å^3^)	1954.23 (6)	2309.45 (14)
*Z*	4	4
Radiation type	Cu *K*α	Cu *K*α
μ (mm^−1^)	2.70	2.35
Crystal size (mm)	0.15 × 0.10 × 0.08	0.11 × 0.07 × 0.02

Data collection		
Diffractometer	Bruker D8 Venture Diffractometer	Bruker D8 Venture Diffractometer
Absorption correction	Multi-scan (*SADABS*; Bruker, 2016 ▸)	Multi-scan (*SADABS*; Bruker, 2016 ▸)
*T*_min_, *T*_max_	0.589, 0.753	0.604, 0.753
No. of measured, independent and observed [*I* > 2σ(*I*)] reflections	44325, 3597, 2964	39443, 4270, 2098
*R*_int_	0.049	0.155
(sin θ/λ)_max_ (Å^−1^)	0.604	0.605

Refinement		
*R*[*F* ^2^ > 2σ(*F* ^2^)], *wR*(*F* ^2^), *S*	0.042, 0.115, 1.03	0.066, 0.232, 1.00
No. of reflections	3597	4270
No. of parameters	272	291
No. of restraints	11	0
H-atom treatment	H atoms treated by a mixture of independent and constrained refinement	H-atom parameters constrained
Δρ_max_, Δρ_min_ (e Å^−3^)	0.21, −0.28	0.33, −0.38

Computer programs: *APEX2* and *SAINT* (Bruker, 2016 ▸), *SHELXS2018/3* (Sheldrick, 2008 ▸), *SHELXL2018/3* (Sheldrick, 2015 ▸), *ORTEP-3 for Windows* and *WinGX* (Farrugia, 2012 ▸), *Mercury* (Macrae *et al.*, 2020 ▸), *publCIF* (Westrip, 2010 ▸) and *PLATON* (Spek, 2020 ▸).

## supplementary crystallographic information

### (E)-N-{2-[2-(Benzo[b]thiophen-2-yl)ethenyl]-5-fluorophenyl}benzenesulfonamide (I) Crystal data

**Table:**

C_22_H_16_FNO_2_S_2_	*F*(000) = 848
*M**_r_* = 409.48	*D*_x_ = 1.392 Mg m^−^^3^
Monoclinic, *P*2_1_/*c*	Cu *K*α radiation, λ = 1.54178 Å
*a* = 7.9588 (1) Å	Cell parameters from 9457 reflections
*b* = 25.9840 (4) Å	θ = 3.4–68.6°
*c* = 9.5178 (2) Å	µ = 2.70 mm^−^^1^
β = 96.853 (1)°	*T* = 297 K
*V* = 1954.23 (6) Å^3^	Block, brown
*Z* = 4	0.15 × 0.10 × 0.08 mm

### (E)-N-{2-[2-(Benzo[b]thiophen-2-yl)ethenyl]-5-fluorophenyl}benzenesulfonamide (I) Data collection

**Table:**

Bruker D8 Venture Diffractometer	2964 reflections with *I* > 2σ(*I*)
Radiation source: micro focus sealed tube	*R*_int_ = 0.049
ω and φ scans	θ_max_ = 68.7°, θ_min_ = 3.4°
Absorption correction: multi-scan (*SADABS*; Bruker, 2016)	*h* = −9→9
*T*_min_ = 0.589, *T*_max_ = 0.753	*k* = −31→31
44325 measured reflections	*l* = −11→11
3597 independent reflections	

### (E)-N-{2-[2-(Benzo[b]thiophen-2-yl)ethenyl]-5-fluorophenyl}benzenesulfonamide (I) Refinement

**Table:**

Refinement on *F*^2^	11 restraints
Least-squares matrix: full	Hydrogen site location: mixed
*R*[*F*^2^ > 2σ(*F*^2^)] = 0.042	H atoms treated by a mixture of independent and constrained refinement
*wR*(*F*^2^) = 0.115	*w* = 1/[σ^2^(*F*_o_^2^) + (0.0496*P*)^2^ + 0.7846*P*] where *P* = (*F*_o_^2^ + 2*F*_c_^2^)/3
*S* = 1.03	(Δ/σ)_max_ = 0.001
3597 reflections	Δρ_max_ = 0.21 e Å^−^^3^
272 parameters	Δρ_min_ = −0.28 e Å^−^^3^

### (E)-N-{2-[2-(Benzo[b]thiophen-2-yl)ethenyl]-5-fluorophenyl}benzenesulfonamide (I) Special details

**Table:**

Geometry. All esds (except the esd in the dihedral angle between two l.s. planes) are estimated using the full covariance matrix. The cell esds are taken into account individually in the estimation of esds in distances, angles and torsion angles; correlations between esds in cell parameters are only used when they are defined by crystal symmetry. An approximate (isotropic) treatment of cell esds is used for estimating esds involving l.s. planes.

### (E)-N-{2-[2-(Benzo[b]thiophen-2-yl)ethenyl]-5-fluorophenyl}benzenesulfonamide (I) Fractional atomic coordinates and isotropic or equivalent isotropic displacement parameters (Å^2^)

**Table:**

	*x*	*y*	*z*	*U*_iso_*/*U*_eq_	Occ. (<1)
C1	0.7141 (2)	0.64416 (6)	0.53276 (16)	0.0782 (7)	
C2	0.6743 (3)	0.69557 (7)	0.5055 (2)	0.1055 (10)	
H2	0.611424	0.704923	0.420704	0.127*	
C3	0.7286 (3)	0.73300 (5)	0.6050 (3)	0.1127 (11)	
H3	0.701996	0.767393	0.586823	0.135*	
C4	0.8226 (3)	0.71902 (7)	0.7318 (2)	0.1109 (11)	
H4	0.858902	0.744059	0.798407	0.133*	
C5	0.8623 (3)	0.66761 (8)	0.75906 (17)	0.1018 (9)	
H5	0.925238	0.658254	0.843872	0.122*	
C6	0.8081 (2)	0.63018 (5)	0.65953 (18)	0.0788 (7)	
S1'	0.8669 (7)	0.56832 (19)	0.7105 (5)	0.0855 (14)	0.243 (4)
C7'	0.709 (3)	0.5902 (6)	0.4679 (19)	0.103 (8)	0.243 (4)
H7'	0.649984	0.584659	0.378833	0.124*	0.243 (4)
C8'	0.790 (5)	0.5504 (7)	0.539 (2)	0.051 (5)	0.243 (4)
S1	0.6512 (2)	0.59509 (5)	0.41950 (14)	0.0758 (4)	0.757 (4)
C7	0.8282 (9)	0.5758 (2)	0.6526 (7)	0.0900 (18)	0.757 (4)
H7	0.893320	0.557894	0.724278	0.108*	0.757 (4)
C8	0.7498 (15)	0.5511 (3)	0.5389 (9)	0.067 (3)	0.757 (4)
C9	0.7644 (3)	0.49638 (9)	0.5064 (3)	0.0754 (6)	
H9	0.808242	0.474482	0.579001	0.090*	
C10	0.6922 (3)	0.47259 (9)	0.3953 (3)	0.0741 (6)	
H10	0.632873	0.492988	0.325994	0.089*	
C11	0.6936 (3)	0.41711 (8)	0.3671 (2)	0.0673 (6)	
C12	0.7752 (4)	0.38326 (10)	0.4678 (3)	0.0976 (9)	
H12	0.829122	0.396833	0.551708	0.117*	
C13	0.7790 (5)	0.33108 (10)	0.4481 (3)	0.1019 (10)	
H13	0.835045	0.309475	0.516146	0.122*	
C14	0.6980 (4)	0.31212 (9)	0.3256 (3)	0.0837 (7)	
C15	0.6143 (3)	0.34215 (8)	0.2231 (3)	0.0711 (6)	
H15	0.557999	0.327509	0.141557	0.085*	
C16	0.6145 (3)	0.39533 (7)	0.2428 (2)	0.0578 (5)	
C17	0.6983 (2)	0.40434 (7)	−0.0853 (2)	0.0545 (5)	
C18	0.8053 (3)	0.44596 (9)	−0.0912 (3)	0.0739 (6)	
H18	0.770751	0.478886	−0.069161	0.089*	
C19	0.9636 (3)	0.43766 (13)	−0.1303 (3)	0.0927 (8)	
H19	1.037422	0.465199	−0.134587	0.111*	
C20	1.0139 (3)	0.38892 (15)	−0.1632 (3)	0.0947 (9)	
H20	1.121259	0.383777	−0.189882	0.114*	
C21	0.9072 (4)	0.34806 (12)	−0.1569 (3)	0.0888 (8)	
H21	0.942294	0.315210	−0.179119	0.107*	
C22	0.7481 (3)	0.35538 (9)	−0.1179 (2)	0.0689 (6)	
H22	0.675016	0.327657	−0.113642	0.083*	
N1	0.5283 (2)	0.42741 (6)	0.1368 (2)	0.0616 (4)	
O1	0.40697 (18)	0.36642 (5)	−0.05058 (18)	0.0706 (4)	
O2	0.42235 (19)	0.45927 (5)	−0.09632 (17)	0.0674 (4)	
F1	0.6977 (3)	0.26050 (5)	0.3036 (2)	0.1171 (6)	
S2	0.49738 (6)	0.41360 (2)	−0.03136 (6)	0.05557 (17)	
H1N	0.545 (3)	0.4587 (6)	0.147 (2)	0.067*	

### (E)-N-{2-[2-(Benzo[b]thiophen-2-yl)ethenyl]-5-fluorophenyl}benzenesulfonamide (I) Atomic displacement parameters (Å^2^)

**Table:**

	*U*^11^	*U*^22^	*U*^33^	*U*^12^	*U*^13^	*U*^23^
C1	0.0989 (18)	0.0723 (15)	0.0687 (14)	−0.0213 (13)	0.0318 (13)	−0.0024 (12)
C2	0.132 (3)	0.0839 (19)	0.103 (2)	−0.0145 (18)	0.0231 (19)	0.0204 (17)
C3	0.135 (3)	0.0621 (16)	0.149 (3)	−0.0154 (17)	0.052 (3)	−0.0014 (19)
C4	0.135 (3)	0.081 (2)	0.126 (3)	−0.0312 (19)	0.054 (2)	−0.0337 (19)
C5	0.124 (2)	0.099 (2)	0.0829 (18)	−0.0240 (19)	0.0139 (17)	−0.0201 (16)
C6	0.0929 (17)	0.0706 (15)	0.0766 (16)	−0.0123 (13)	0.0252 (13)	−0.0080 (12)
S1'	0.104 (3)	0.077 (2)	0.074 (3)	−0.0128 (19)	0.004 (2)	−0.0059 (19)
C7'	0.127 (16)	0.121 (16)	0.059 (10)	−0.069 (13)	0.001 (8)	−0.016 (10)
C8'	0.045 (12)	0.060 (8)	0.050 (8)	−0.004 (5)	0.017 (5)	0.001 (6)
S1	0.1095 (10)	0.0607 (5)	0.0576 (8)	−0.0079 (5)	0.0109 (6)	−0.0006 (4)
C7	0.111 (4)	0.077 (3)	0.076 (4)	0.003 (2)	−0.010 (3)	0.000 (3)
C8	0.065 (6)	0.067 (3)	0.071 (3)	−0.009 (2)	0.017 (2)	−0.003 (2)
C9	0.0896 (17)	0.0647 (14)	0.0712 (14)	0.0018 (12)	0.0068 (12)	0.0001 (11)
C10	0.1028 (18)	0.0551 (12)	0.0632 (13)	−0.0022 (12)	0.0055 (12)	0.0060 (10)
C11	0.0864 (15)	0.0516 (11)	0.0647 (13)	0.0000 (10)	0.0123 (11)	0.0086 (10)
C12	0.142 (3)	0.0674 (16)	0.0772 (17)	−0.0011 (16)	−0.0111 (17)	0.0139 (13)
C13	0.141 (3)	0.0651 (16)	0.095 (2)	0.0093 (16)	−0.0046 (19)	0.0296 (15)
C14	0.114 (2)	0.0459 (12)	0.0930 (19)	0.0051 (12)	0.0186 (16)	0.0146 (12)
C15	0.0888 (16)	0.0472 (11)	0.0783 (15)	0.0022 (11)	0.0142 (12)	0.0065 (10)
C16	0.0645 (12)	0.0457 (10)	0.0658 (12)	0.0025 (9)	0.0186 (10)	0.0083 (9)
C17	0.0545 (10)	0.0502 (10)	0.0561 (11)	−0.0008 (8)	−0.0041 (8)	−0.0033 (8)
C18	0.0654 (13)	0.0673 (14)	0.0871 (16)	−0.0139 (11)	0.0014 (12)	−0.0091 (12)
C19	0.0671 (15)	0.116 (2)	0.0935 (19)	−0.0253 (16)	0.0020 (14)	−0.0039 (17)
C20	0.0562 (14)	0.147 (3)	0.0796 (17)	0.0120 (17)	0.0026 (12)	−0.0036 (18)
C21	0.0794 (17)	0.096 (2)	0.0904 (19)	0.0269 (15)	0.0098 (14)	−0.0087 (15)
C22	0.0724 (14)	0.0582 (12)	0.0752 (14)	0.0077 (10)	0.0049 (11)	−0.0050 (11)
N1	0.0745 (11)	0.0401 (8)	0.0700 (11)	0.0070 (8)	0.0081 (9)	0.0009 (8)
O1	0.0613 (8)	0.0459 (7)	0.1008 (12)	−0.0087 (6)	−0.0061 (8)	−0.0028 (7)
O2	0.0711 (9)	0.0437 (7)	0.0826 (10)	0.0098 (6)	−0.0111 (7)	0.0015 (7)
F1	0.1731 (17)	0.0454 (8)	0.1300 (14)	0.0126 (9)	0.0067 (12)	0.0189 (8)
S2	0.0546 (3)	0.0379 (2)	0.0718 (3)	0.00112 (18)	−0.0029 (2)	−0.0018 (2)

### (E)-N-{2-[2-(Benzo[b]thiophen-2-yl)ethenyl]-5-fluorophenyl}benzenesulfonamide (I) Geometric parameters (Å, º)

**Table:**

C1—C2	1.3900	C11—C12	1.402 (3)
C1—C6	1.3900	C12—C13	1.369 (4)
C1—C7'	1.531 (14)	C12—H12	0.9300
C1—S1	1.7058 (18)	C13—C14	1.356 (4)
C2—C3	1.3900	C13—H13	0.9300
C2—H2	0.9300	C14—F1	1.358 (3)
C3—C4	1.3900	C14—C15	1.360 (3)
C3—H3	0.9300	C15—C16	1.394 (3)
C4—C5	1.3900	C15—H15	0.9300
C4—H4	0.9300	C16—N1	1.421 (3)
C5—C6	1.3900	C17—C22	1.379 (3)
C5—H5	0.9300	C17—C18	1.381 (3)
C6—C7	1.426 (6)	C17—S2	1.754 (2)
C6—S1'	1.727 (5)	C18—C19	1.373 (4)
S1'—C8'	1.738 (17)	C18—H18	0.9300
C7'—C8'	1.356 (18)	C19—C20	1.375 (4)
C7'—H7'	0.9300	C19—H19	0.9300
C8'—C9	1.446 (17)	C20—C21	1.365 (4)
S1—C8	1.732 (7)	C20—H20	0.9300
C7—C8	1.345 (8)	C21—C22	1.375 (4)
C7—H7	0.9300	C21—H21	0.9300
C8—C9	1.463 (7)	C22—H22	0.9300
C9—C10	1.298 (3)	N1—S2	1.6301 (19)
C9—H9	0.9300	N1—H1N	0.828 (16)
C10—C11	1.467 (3)	O1—S2	1.4222 (14)
C10—H10	0.9300	O2—S2	1.4348 (14)
C11—C16	1.393 (3)		
			
C2—C1—C6	120.0	C16—C11—C10	122.8 (2)
C2—C1—C7'	144.2 (7)	C12—C11—C10	120.3 (2)
C6—C1—C7'	95.5 (7)	C13—C12—C11	122.9 (3)
C2—C1—S1	123.88 (12)	C13—C12—H12	118.5
C6—C1—S1	116.11 (12)	C11—C12—H12	118.5
C3—C2—C1	120.0	C14—C13—C12	117.5 (3)
C3—C2—H2	120.0	C14—C13—H13	121.3
C1—C2—H2	120.0	C12—C13—H13	121.3
C2—C3—C4	120.0	C13—C14—F1	118.9 (2)
C2—C3—H3	120.0	C13—C14—C15	123.4 (2)
C4—C3—H3	120.0	F1—C14—C15	117.7 (3)
C5—C4—C3	120.0	C14—C15—C16	118.6 (2)
C5—C4—H4	120.0	C14—C15—H15	120.7
C3—C4—H4	120.0	C16—C15—H15	120.7
C4—C5—C6	120.0	C11—C16—C15	120.7 (2)
C4—C5—H5	120.0	C11—C16—N1	119.74 (18)
C6—C5—H5	120.0	C15—C16—N1	119.6 (2)
C5—C6—C1	120.0	C22—C17—C18	121.2 (2)
C5—C6—C7	134.3 (3)	C22—C17—S2	119.30 (17)
C1—C6—C7	105.7 (3)	C18—C17—S2	119.49 (17)
C5—C6—S1'	114.1 (2)	C19—C18—C17	118.5 (2)
C1—C6—S1'	125.9 (2)	C19—C18—H18	120.7
C6—S1'—C8'	86.1 (6)	C17—C18—H18	120.7
C8'—C7'—C1	120.6 (13)	C18—C19—C20	120.5 (3)
C8'—C7'—H7'	119.7	C18—C19—H19	119.7
C1—C7'—H7'	119.7	C20—C19—H19	119.7
C7'—C8'—C9	126.0 (15)	C21—C20—C19	120.5 (3)
C7'—C8'—S1'	110.9 (13)	C21—C20—H20	119.8
C9—C8'—S1'	119.1 (13)	C19—C20—H20	119.8
C1—S1—C8	90.1 (2)	C20—C21—C22	120.1 (3)
C8—C7—C6	117.7 (6)	C20—C21—H21	119.9
C8—C7—H7	121.1	C22—C21—H21	119.9
C6—C7—H7	121.1	C21—C22—C17	119.2 (2)
C7—C8—C9	126.3 (6)	C21—C22—H22	120.4
C7—C8—S1	110.1 (5)	C17—C22—H22	120.4
C9—C8—S1	122.9 (5)	C16—N1—S2	124.85 (14)
C10—C9—C8'	132.4 (8)	C16—N1—H1N	116.0 (16)
C10—C9—C8	126.6 (4)	S2—N1—H1N	109.5 (16)
C10—C9—H9	113.8	O1—S2—O2	118.64 (9)
C8'—C9—H9	113.8	O1—S2—N1	109.32 (10)
C9—C10—C11	127.2 (2)	O2—S2—N1	104.32 (9)
C9—C10—H10	116.4	O1—S2—C17	108.01 (9)
C11—C10—H10	116.4	O2—S2—C17	109.55 (10)
C16—C11—C12	116.8 (2)	N1—S2—C17	106.35 (9)
			
C6—C1—C2—C3	0.0	C8'—C9—C10—C11	171 (2)
C7'—C1—C2—C3	−171.5 (14)	C8—C9—C10—C11	−174.3 (7)
S1—C1—C2—C3	178.91 (16)	C9—C10—C11—C16	−178.7 (3)
C1—C2—C3—C4	0.0	C9—C10—C11—C12	2.1 (4)
C2—C3—C4—C5	0.0	C16—C11—C12—C13	−0.1 (5)
C3—C4—C5—C6	0.0	C10—C11—C12—C13	179.2 (3)
C4—C5—C6—C1	0.0	C11—C12—C13—C14	−0.7 (5)
C4—C5—C6—C7	178.8 (4)	C12—C13—C14—F1	−179.2 (3)
C4—C5—C6—S1'	−179.6 (2)	C12—C13—C14—C15	−0.1 (5)
C2—C1—C6—C5	0.0	C13—C14—C15—C16	1.5 (4)
C7'—C1—C6—C5	175.0 (8)	F1—C14—C15—C16	−179.3 (2)
S1—C1—C6—C5	−178.99 (15)	C12—C11—C16—C15	1.6 (4)
C2—C1—C6—C7	−179.1 (3)	C10—C11—C16—C15	−177.7 (2)
S1—C1—C6—C7	1.9 (3)	C12—C11—C16—N1	179.5 (2)
C2—C1—C6—S1'	179.5 (3)	C10—C11—C16—N1	0.2 (3)
C7'—C1—C6—S1'	−5.5 (8)	C14—C15—C16—C11	−2.3 (4)
C5—C6—S1'—C8'	−171.8 (13)	C14—C15—C16—N1	179.8 (2)
C1—C6—S1'—C8'	8.7 (14)	C22—C17—C18—C19	−0.1 (4)
C2—C1—C7'—C8'	171 (2)	S2—C17—C18—C19	178.41 (19)
C6—C1—C7'—C8'	−2 (3)	C17—C18—C19—C20	0.2 (4)
C1—C7'—C8'—C9	165 (2)	C18—C19—C20—C21	−0.2 (4)
C1—C7'—C8'—S1'	8 (4)	C19—C20—C21—C22	0.2 (4)
C6—S1'—C8'—C7'	−8 (2)	C20—C21—C22—C17	−0.1 (4)
C6—S1'—C8'—C9	−167 (3)	C18—C17—C22—C21	0.0 (4)
C2—C1—S1—C8	−178.8 (5)	S2—C17—C22—C21	−178.48 (19)
C6—C1—S1—C8	0.1 (5)	C11—C16—N1—S2	153.99 (18)
C5—C6—C7—C8	177.3 (7)	C15—C16—N1—S2	−28.1 (3)
C1—C6—C7—C8	−3.8 (9)	C16—N1—S2—O1	58.1 (2)
C6—C7—C8—C9	175.1 (8)	C16—N1—S2—O2	−174.05 (17)
C6—C7—C8—S1	4.0 (11)	C16—N1—S2—C17	−58.28 (19)
C1—S1—C8—C7	−2.2 (8)	C22—C17—S2—O1	−8.2 (2)
C1—S1—C8—C9	−173.7 (9)	C18—C17—S2—O1	173.24 (18)
C7'—C8'—C9—C10	15 (5)	C22—C17—S2—O2	−138.77 (17)
S1'—C8'—C9—C10	170.8 (10)	C18—C17—S2—O2	42.7 (2)
C7—C8—C9—C10	−178.3 (8)	C22—C17—S2—N1	109.03 (18)
S1—C8—C9—C10	−8.4 (13)	C18—C17—S2—N1	−69.51 (19)

### (E)-N-{2-[2-(Benzo[b]thiophen-2-yl)ethenyl]-5-fluorophenyl}benzenesulfonamide (I) Hydrogen-bond geometry (Å, º)

**Table:**

*D*—H···*A*	*D*—H	H···*A*	*D*···*A*	*D*—H···*A*
C15—H15···O1	0.93	2.30	2.980 (3)	130
C10—H10···02^i^	0.93	2.51	3.383 (3)	157
N1—H1*N*···O2^i^	0.83 (2)	2.21 (2)	3.001 (2)	161 (2)
C22—H22···F1^ii^	0.93	2.44	3.116 (3)	130

Symmetry codes: (i) −*x*+1, −*y*+1, −*z*; (ii) *x*, −*y*+1/2, *z*−1/2.

### (E)-N-{2-[2-(Benzo[b]thiophen-2-yl)ethenyl]-5-fluorophenyl}-N-(but-2-yn-1-yl)benzenesulfonamide (II) Crystal data

**Table:**

C_26_H_20_FNO_2_S_2_	*F*(000) = 960
*M**_r_* = 461.55	*D*_x_ = 1.327 Mg m^−^^3^
Monoclinic, *P*2_1_/*c*	Cu *K*α radiation, λ = 1.54178 Å
*a* = 9.3517 (3) Å	Cell parameters from 3616 reflections
*b* = 31.7075 (11) Å	θ = 2.8–65.4°
*c* = 8.6063 (3) Å	µ = 2.35 mm^−^^1^
β = 115.179 (2)°	*T* = 297 K
*V* = 2309.45 (14) Å^3^	Solid, white
*Z* = 4	0.11 × 0.07 × 0.02 mm

### (E)-N-{2-[2-(Benzo[b]thiophen-2-yl)ethenyl]-5-fluorophenyl}-N-(but-2-yn-1-yl)benzenesulfonamide (II) Data collection

**Table:**

Bruker D8 Venture Diffractometer	2098 reflections with *I* > 2σ(*I*)
Radiation source: micro focus sealed tube	*R*_int_ = 0.155
ω and φ scans	θ_max_ = 68.8°, θ_min_ = 5.2°
Absorption correction: multi-scan (*SADABS*; Bruker, 2016)	*h* = −11→11
*T*_min_ = 0.604, *T*_max_ = 0.753	*k* = −37→38
39443 measured reflections	*l* = −10→10
4270 independent reflections	

### (E)-N-{2-[2-(Benzo[b]thiophen-2-yl)ethenyl]-5-fluorophenyl}-N-(but-2-yn-1-yl)benzenesulfonamide (II) Refinement

**Table:**

Refinement on *F*^2^	Hydrogen site location: inferred from neighbouring sites
Least-squares matrix: full	H-atom parameters constrained
*R*[*F*^2^ > 2σ(*F*^2^)] = 0.066	*w* = 1/[σ^2^(*F*_o_^2^) + (0.1325*P*)^2^] where *P* = (*F*_o_^2^ + 2*F*_c_^2^)/3
*wR*(*F*^2^) = 0.232	(Δ/σ)_max_ < 0.001
*S* = 1.00	Δρ_max_ = 0.33 e Å^−^^3^
4270 reflections	Δρ_min_ = −0.37 e Å^−^^3^
291 parameters	Extinction correction: SHELXL-2018/3 (Sheldrick 2015), Fc^*^=kFc[1+0.001xFc^2^λ^3^/sin(2θ)]^-1/4^
0 restraints	Extinction coefficient: 0.0040 (6)

### (E)-N-{2-[2-(Benzo[b]thiophen-2-yl)ethenyl]-5-fluorophenyl}-N-(but-2-yn-1-yl)benzenesulfonamide (II) Special details

**Table:**

Geometry. All esds (except the esd in the dihedral angle between two l.s. planes) are estimated using the full covariance matrix. The cell esds are taken into account individually in the estimation of esds in distances, angles and torsion angles; correlations between esds in cell parameters are only used when they are defined by crystal symmetry. An approximate (isotropic) treatment of cell esds is used for estimating esds involving l.s. planes.

### (E)-N-{2-[2-(Benzo[b]thiophen-2-yl)ethenyl]-5-fluorophenyl}-N-(but-2-yn-1-yl)benzenesulfonamide (II) Fractional atomic coordinates and isotropic or equivalent isotropic displacement parameters (Å^2^)

**Table:**

	*x*	*y*	*z*	*U*_iso_*/*U*_eq_	
C1	0.2549 (6)	0.32808 (15)	−0.0285 (6)	0.0669 (12)	
C2	0.2360 (6)	0.33934 (17)	−0.1918 (6)	0.0778 (14)	
H2	0.311900	0.355578	−0.207042	0.093*	
C3	0.1029 (7)	0.32600 (17)	−0.3309 (7)	0.0825 (15)	
H3	0.089065	0.333320	−0.441041	0.099*	
C4	−0.0110 (6)	0.30184 (16)	−0.3096 (6)	0.0763 (14)	
H4	−0.100010	0.293249	−0.405677	0.092*	
C5	0.0054 (6)	0.29044 (15)	−0.1498 (6)	0.0722 (13)	
H5	−0.071951	0.274308	−0.137015	0.087*	
C6	0.1408 (5)	0.30337 (14)	−0.0042 (5)	0.0626 (11)	
C7	0.1836 (6)	0.29535 (14)	0.1732 (6)	0.0673 (12)	
H7	0.122120	0.279321	0.212083	0.081*	
C8	0.3227 (5)	0.31338 (13)	0.2798 (5)	0.0609 (11)	
C9	0.3969 (6)	0.31034 (15)	0.4648 (6)	0.0682 (12)	
H9	0.350312	0.292242	0.514847	0.082*	
C10	0.5265 (6)	0.33107 (14)	0.5712 (6)	0.0633 (12)	
H10	0.571084	0.350331	0.523168	0.076*	
C11	0.6030 (5)	0.32555 (13)	0.7581 (5)	0.0586 (11)	
C12	0.5852 (6)	0.28800 (14)	0.8336 (6)	0.0659 (12)	
H12	0.521274	0.266825	0.763600	0.079*	
C13	0.6595 (6)	0.28137 (15)	1.0084 (6)	0.0702 (13)	
H13	0.646816	0.256223	1.056735	0.084*	
C14	0.7527 (6)	0.31322 (16)	1.1085 (6)	0.0718 (13)	
C15	0.7743 (6)	0.35085 (15)	1.0435 (6)	0.0694 (13)	
H15	0.836300	0.371959	1.115842	0.083*	
C16	0.7016 (5)	0.35672 (13)	0.8677 (5)	0.0585 (11)	
C17	0.6526 (6)	0.45543 (14)	0.9821 (6)	0.0688 (13)	
C18	0.5818 (8)	0.43930 (18)	1.0789 (7)	0.0899 (17)	
H18	0.505581	0.418355	1.033546	0.108*	
C19	0.6226 (9)	0.4539 (2)	1.2444 (9)	0.106 (2)	
H19	0.574503	0.442786	1.310278	0.128*	
C20	0.7344 (9)	0.4848 (2)	1.3093 (9)	0.109 (2)	
H20	0.762946	0.494415	1.420623	0.131*	
C21	0.8042 (9)	0.5017 (2)	1.2151 (9)	0.109 (2)	
H21	0.878624	0.523052	1.260868	0.130*	
C22	0.7647 (7)	0.48710 (16)	1.0491 (7)	0.0872 (16)	
H22	0.812980	0.498474	0.983836	0.105*	
C23	0.8877 (6)	0.40504 (16)	0.8143 (7)	0.0763 (14)	
H23A	0.925473	0.380835	0.773623	0.092*	
H23B	0.882648	0.428675	0.740500	0.092*	
C24	1.0039 (6)	0.41494 (15)	0.9891 (7)	0.0764 (14)	
C25	1.0953 (7)	0.42202 (17)	1.1328 (8)	0.0886 (17)	
C26	1.2031 (8)	0.4310 (2)	1.3114 (8)	0.117 (2)	
H26A	1.143024	0.438114	1.374590	0.175*	
H26B	1.270375	0.454214	1.315234	0.175*	
H26C	1.266516	0.406545	1.361637	0.175*	
N1	0.7255 (4)	0.39583 (10)	0.7947 (5)	0.0613 (10)	
O1	0.4505 (4)	0.41733 (11)	0.7156 (4)	0.0851 (11)	
O2	0.6400 (5)	0.46681 (11)	0.6790 (5)	0.0949 (12)	
S1	0.40923 (16)	0.34048 (5)	0.16627 (16)	0.0776 (5)	
S2	0.60508 (16)	0.43535 (4)	0.77698 (15)	0.0715 (4)	
F1	0.8245 (4)	0.30760 (10)	1.2817 (3)	0.0982 (10)	

### (E)-N-{2-[2-(Benzo[b]thiophen-2-yl)ethenyl]-5-fluorophenyl}-N-(but-2-yn-1-yl)benzenesulfonamide (II) Atomic displacement parameters (Å^2^)

**Table:**

	*U*^11^	*U*^22^	*U*^33^	*U*^12^	*U*^13^	*U*^23^
C1	0.060 (3)	0.078 (3)	0.063 (3)	−0.003 (2)	0.026 (2)	−0.005 (2)
C2	0.073 (3)	0.097 (4)	0.066 (3)	−0.005 (3)	0.033 (3)	0.000 (3)
C3	0.080 (4)	0.107 (4)	0.059 (3)	0.005 (3)	0.029 (3)	−0.003 (3)
C4	0.074 (3)	0.089 (3)	0.062 (3)	0.001 (3)	0.025 (3)	−0.013 (2)
C5	0.063 (3)	0.079 (3)	0.066 (3)	−0.008 (2)	0.019 (2)	−0.013 (2)
C6	0.060 (3)	0.071 (3)	0.056 (2)	−0.001 (2)	0.024 (2)	−0.008 (2)
C7	0.065 (3)	0.072 (3)	0.065 (3)	−0.015 (2)	0.028 (2)	−0.005 (2)
C8	0.063 (3)	0.064 (2)	0.057 (2)	−0.005 (2)	0.027 (2)	−0.003 (2)
C9	0.068 (3)	0.071 (3)	0.061 (3)	−0.007 (2)	0.023 (2)	−0.004 (2)
C10	0.064 (3)	0.065 (3)	0.059 (2)	−0.001 (2)	0.025 (2)	0.000 (2)
C11	0.057 (3)	0.061 (2)	0.057 (2)	0.000 (2)	0.024 (2)	0.0004 (19)
C12	0.068 (3)	0.064 (3)	0.066 (3)	−0.003 (2)	0.029 (2)	0.000 (2)
C13	0.076 (3)	0.070 (3)	0.067 (3)	0.000 (3)	0.034 (3)	0.007 (2)
C14	0.079 (3)	0.081 (3)	0.049 (2)	0.007 (3)	0.021 (2)	0.009 (2)
C15	0.070 (3)	0.070 (3)	0.060 (3)	−0.001 (2)	0.020 (2)	−0.001 (2)
C16	0.062 (3)	0.056 (2)	0.057 (2)	0.002 (2)	0.025 (2)	0.0017 (18)
C17	0.076 (3)	0.063 (3)	0.070 (3)	0.003 (2)	0.033 (3)	−0.001 (2)
C18	0.107 (5)	0.085 (3)	0.081 (4)	−0.009 (3)	0.044 (3)	−0.010 (3)
C19	0.132 (6)	0.106 (4)	0.096 (5)	0.003 (4)	0.063 (4)	−0.003 (4)
C20	0.134 (6)	0.110 (5)	0.079 (4)	0.016 (5)	0.042 (4)	−0.019 (4)
C21	0.119 (5)	0.096 (4)	0.099 (5)	−0.019 (4)	0.034 (4)	−0.035 (4)
C22	0.099 (4)	0.072 (3)	0.088 (4)	−0.016 (3)	0.038 (3)	−0.014 (3)
C23	0.074 (3)	0.076 (3)	0.081 (3)	−0.009 (3)	0.035 (3)	−0.005 (2)
C24	0.068 (3)	0.068 (3)	0.090 (4)	−0.008 (3)	0.031 (3)	−0.005 (3)
C25	0.081 (4)	0.075 (3)	0.101 (4)	−0.008 (3)	0.030 (3)	−0.008 (3)
C26	0.102 (5)	0.123 (5)	0.093 (4)	−0.012 (4)	0.010 (4)	−0.015 (4)
N1	0.064 (2)	0.0555 (19)	0.061 (2)	−0.0017 (17)	0.0238 (18)	0.0022 (16)
O1	0.062 (2)	0.084 (2)	0.088 (2)	0.0006 (18)	0.0117 (18)	−0.0075 (18)
O2	0.139 (3)	0.0680 (19)	0.077 (2)	0.000 (2)	0.045 (2)	0.0174 (17)
S1	0.0655 (8)	0.0998 (9)	0.0656 (7)	−0.0180 (7)	0.0261 (6)	−0.0056 (6)
S2	0.0822 (9)	0.0616 (6)	0.0625 (7)	0.0020 (6)	0.0229 (6)	0.0030 (5)
F1	0.114 (2)	0.107 (2)	0.0552 (16)	−0.0063 (19)	0.0187 (16)	0.0166 (15)

### (E)-N-{2-[2-(Benzo[b]thiophen-2-yl)ethenyl]-5-fluorophenyl}-N-(but-2-yn-1-yl)benzenesulfonamide (II) Geometric parameters (Å, º)

**Table:**

C1—C2	1.387 (7)	C15—C16	1.382 (6)
C1—C6	1.409 (7)	C15—H15	0.9300
C1—S1	1.729 (5)	C16—N1	1.450 (5)
C2—C3	1.376 (7)	C17—C18	1.365 (7)
C2—H2	0.9300	C17—C22	1.387 (7)
C3—C4	1.386 (7)	C17—S2	1.747 (5)
C3—H3	0.9300	C18—C19	1.388 (8)
C4—C5	1.367 (7)	C18—H18	0.9300
C4—H4	0.9300	C19—C20	1.368 (9)
C5—C6	1.411 (6)	C19—H19	0.9300
C5—H5	0.9300	C20—C21	1.349 (9)
C6—C7	1.426 (6)	C20—H20	0.9300
C7—C8	1.358 (6)	C21—C22	1.394 (8)
C7—H7	0.9300	C21—H21	0.9300
C8—C9	1.444 (6)	C22—H22	0.9300
C8—S1	1.738 (5)	C23—C24	1.467 (7)
C9—C10	1.340 (6)	C23—N1	1.483 (6)
C9—H9	0.9300	C23—H23A	0.9700
C10—C11	1.466 (6)	C23—H23B	0.9700
C10—H10	0.9300	C24—C25	1.188 (7)
C11—C12	1.400 (6)	C25—C26	1.464 (8)
C11—C16	1.406 (6)	C26—H26A	0.9600
C12—C13	1.378 (6)	C26—H26B	0.9600
C12—H12	0.9300	C26—H26C	0.9600
C13—C14	1.372 (7)	N1—S2	1.648 (4)
C13—H13	0.9300	O1—S2	1.431 (4)
C14—F1	1.361 (5)	O2—S2	1.431 (4)
C14—C15	1.369 (7)		
			
C2—C1—C6	121.0 (4)	C15—C16—C11	121.1 (4)
C2—C1—S1	128.2 (4)	C15—C16—N1	119.7 (4)
C6—C1—S1	110.8 (3)	C11—C16—N1	119.2 (4)
C3—C2—C1	118.7 (5)	C18—C17—C22	119.6 (5)
C3—C2—H2	120.6	C18—C17—S2	119.9 (4)
C1—C2—H2	120.6	C22—C17—S2	120.4 (4)
C2—C3—C4	121.1 (5)	C17—C18—C19	120.6 (6)
C2—C3—H3	119.4	C17—C18—H18	119.7
C4—C3—H3	119.4	C19—C18—H18	119.7
C5—C4—C3	121.0 (5)	C20—C19—C18	119.1 (7)
C5—C4—H4	119.5	C20—C19—H19	120.4
C3—C4—H4	119.5	C18—C19—H19	120.4
C4—C5—C6	119.4 (5)	C21—C20—C19	121.4 (6)
C4—C5—H5	120.3	C21—C20—H20	119.3
C6—C5—H5	120.3	C19—C20—H20	119.3
C1—C6—C5	118.7 (4)	C20—C21—C22	119.9 (6)
C1—C6—C7	111.9 (4)	C20—C21—H21	120.0
C5—C6—C7	129.5 (5)	C22—C21—H21	120.0
C8—C7—C6	113.6 (4)	C17—C22—C21	119.3 (6)
C8—C7—H7	123.2	C17—C22—H22	120.3
C6—C7—H7	123.2	C21—C22—H22	120.3
C7—C8—C9	126.1 (4)	C24—C23—N1	115.7 (4)
C7—C8—S1	111.7 (3)	C24—C23—H23A	108.3
C9—C8—S1	122.2 (3)	N1—C23—H23A	108.3
C10—C9—C8	126.3 (5)	C24—C23—H23B	108.3
C10—C9—H9	116.9	N1—C23—H23B	108.3
C8—C9—H9	116.9	H23A—C23—H23B	107.4
C9—C10—C11	124.7 (5)	C25—C24—C23	177.7 (6)
C9—C10—H10	117.7	C24—C25—C26	177.9 (7)
C11—C10—H10	117.7	C25—C26—H26A	109.5
C12—C11—C16	117.4 (4)	C25—C26—H26B	109.5
C12—C11—C10	120.8 (4)	H26A—C26—H26B	109.5
C16—C11—C10	121.8 (4)	C25—C26—H26C	109.5
C13—C12—C11	122.1 (4)	H26A—C26—H26C	109.5
C13—C12—H12	118.9	H26B—C26—H26C	109.5
C11—C12—H12	118.9	C16—N1—C23	117.3 (4)
C14—C13—C12	117.6 (4)	C16—N1—S2	117.7 (3)
C14—C13—H13	121.2	C23—N1—S2	119.1 (3)
C12—C13—H13	121.2	C1—S1—C8	92.1 (2)
F1—C14—C15	118.2 (4)	O2—S2—O1	120.6 (2)
F1—C14—C13	118.5 (4)	O2—S2—N1	105.5 (2)
C15—C14—C13	123.3 (4)	O1—S2—N1	105.9 (2)
C14—C15—C16	118.4 (4)	O2—S2—C17	108.5 (2)
C14—C15—H15	120.8	O1—S2—C17	107.5 (2)
C16—C15—H15	120.8	N1—S2—C17	108.3 (2)
			
C6—C1—C2—C3	0.2 (8)	C10—C11—C16—N1	1.5 (7)
S1—C1—C2—C3	180.0 (4)	C22—C17—C18—C19	−0.9 (9)
C1—C2—C3—C4	0.1 (8)	S2—C17—C18—C19	177.7 (5)
C2—C3—C4—C5	−0.1 (8)	C17—C18—C19—C20	0.3 (10)
C3—C4—C5—C6	−0.2 (8)	C18—C19—C20—C21	0.8 (11)
C2—C1—C6—C5	−0.5 (7)	C19—C20—C21—C22	−1.1 (11)
S1—C1—C6—C5	179.7 (4)	C18—C17—C22—C21	0.6 (9)
C2—C1—C6—C7	179.8 (5)	S2—C17—C22—C21	−178.0 (5)
S1—C1—C6—C7	−0.1 (5)	C20—C21—C22—C17	0.5 (10)
C4—C5—C6—C1	0.5 (7)	C15—C16—N1—C23	64.9 (6)
C4—C5—C6—C7	−179.8 (5)	C11—C16—N1—C23	−115.9 (5)
C1—C6—C7—C8	0.6 (6)	C15—C16—N1—S2	−87.9 (5)
C5—C6—C7—C8	−179.0 (5)	C11—C16—N1—S2	91.3 (5)
C6—C7—C8—C9	−179.2 (4)	C24—C23—N1—C16	−69.7 (5)
C6—C7—C8—S1	−0.9 (5)	C24—C23—N1—S2	82.7 (5)
C7—C8—C9—C10	−173.6 (5)	C2—C1—S1—C8	179.8 (5)
S1—C8—C9—C10	8.3 (7)	C6—C1—S1—C8	−0.4 (4)
C8—C9—C10—C11	−176.8 (4)	C7—C8—S1—C1	0.7 (4)
C9—C10—C11—C12	24.6 (7)	C9—C8—S1—C1	179.1 (4)
C9—C10—C11—C16	−158.0 (5)	C16—N1—S2—O2	−171.5 (3)
C16—C11—C12—C13	0.5 (7)	C23—N1—S2—O2	36.2 (4)
C10—C11—C12—C13	178.0 (5)	C16—N1—S2—O1	−42.6 (4)
C11—C12—C13—C14	0.2 (7)	C23—N1—S2—O1	165.2 (3)
C12—C13—C14—F1	178.7 (4)	C16—N1—S2—C17	72.5 (4)
C12—C13—C14—C15	0.4 (8)	C23—N1—S2—C17	−79.8 (4)
F1—C14—C15—C16	−180.0 (4)	C18—C17—S2—O2	156.6 (5)
C13—C14—C15—C16	−1.6 (8)	C22—C17—S2—O2	−24.9 (5)
C14—C15—C16—C11	2.4 (7)	C18—C17—S2—O1	24.6 (5)
C14—C15—C16—N1	−178.4 (4)	C22—C17—S2—O1	−156.9 (4)
C12—C11—C16—C15	−1.8 (7)	C18—C17—S2—N1	−89.4 (5)
C10—C11—C16—C15	−179.3 (4)	C22—C17—S2—N1	89.1 (4)
C12—C11—C16—N1	179.0 (4)		

### (E)-N-{2-[2-(Benzo[b]thiophen-2-yl)ethenyl]-5-fluorophenyl}-N-(but-2-yn-1-yl)benzenesulfonamide (II) Hydrogen-bond geometry (Å, º)

*Cg*1 is the centroid of the C1–C6 ring.

**Table:**

*D*—H···*A*	*D*—H	H···*A*	*D*···*A*	*D*—H···*A*
C2—H2···O1^i^	0.93	2.59	3.483 (7)	162
C4—H4···F1^ii^	0.93	2.52	3.188 (6)	130
C18—H18···S1^iii^	0.93	3.01	3.744 (6)	137
C23—H23*A*···*Cg*1^iv^	0.93	2.69	3.566 (6)	151

Symmetry codes: (i) *x*, *y*, *z*−1; (ii) *x*−1, *y*, *z*−2; (iii) *x*, *y*, *z*+1; (iv) *x*+1, *y*, *z*+1.
